# Some Properties of Vulcanite Rubber

**Published:** 1898-09

**Authors:** S. E. Davenport


### SOME PROPERTIES OF VULCANITE RUBBER.[1]

BY S. E. DAVENPORT, D.D.S., M.D.S.

The onward march of specialism in professions accounts for the entrance upon dental practice of many young men who have not been properly grounded in the making and tempering of instruments, the management of fusible metals, the designing of fixtures and appliances, and the proper use of all compounds and materials which belong more to the laboratory than to the operating-room.

We of the old-fashioned school, who were blessed with preceptors who, though destitute, perhaps, of high-flown theories, were very ingenious and had manipulative ability at their finger ends, were taught to make and use the laboratory adjuncts of our calling before we were allowed to operate upon the natural teeth.

I have felt for some years that there were dangers for our patients connected with this division and subdivision into specialties. The same mind should direct for each patient the strictly operative as well as the prosthetic needs, in order that the best result may be obtained.

Illustrations are frequent of a lack of harmony between partial dentures and important fillings in the same mouth, the plates having been made by one dentist and the fillings by another.

It should be a source of satisfaction to dentists in replying to the inquiries of patients that they have no specialties, but are willing to accept the responsibility of ministering to their patients' needs, whether operating, regulating, or the insertion of artificial dentures.

In accordance, therefore, with the opinion expressed above, that patients who require dental services of a varied character are better served, other things being equal, if those services can be rendered, or at least directed, by one man, I desire to add that the dentist who undertakes to render service of a prosthetic nature needs to possess full knowledge of all materials and methods used in the laboratory, even though he may be a very busy man in his operating-room, and not enter that laboratory except for the purpose of directing the workmen therein.

My observation and experience lead me to conclude that a knowledge of laboratory materials, methods, and appliances is distinctly unfashionable.

---

[1] Read before The New York Institute of Stomatology, June 7, 1898.

I take the liberty this evening of calling attention to some properties of vulcanite rubber which I think are not generally understood.

These characteristics are principally useful when changes are being made in old plates, the additions of new rubber or teeth, or the repairing of fractured plates.

I remember well, when practising in the country about twenty years ago, a man coming into the office with bottles of some fluid, the application of which to a rubber plate, he said, would enable one to stick new rubber to the old without subsequent cleavage.

My father, in whose office I was, expressed considerable amusement over this, for he had been adding new rubber to old without the use of chemicals for many years.

Vulcanite rubber, when forcibly pressed with a hot instrument against a clean, dry surface of rubber already vulcanized, will form, when vulcanized, a perfect union with the old rubber, and will break as quickly at any point as at the junction of the new and the old.

Should a rubber plate need a new block or tooth, the old block or tooth having been removed by the application of heat and the pressure of an instrument, a sharp scraper and engine burs should be used to cut away some of the rubber which held the block in position, particularly the portions in which the pins were embedded.

The new block now being ground and fitted for position, the old rubber is again scraped to insure a clean, dry surface, and small pieces of rubber should be spread upon this surface with a flattened instrument repeatedly heated in a flame. An instrument which is not sufficiently hot sticks to the fresh rubber and causes it to drag. When sufficient rubber is stuck to the surface, the block should be heated and pressed to its place, a cloth protecting the hand. Any rubber which may squeeze out should be trimmed off, the plate invested and vulcanized.

Fractured plates should be brought into proper position, the pieces being held with softened wax on the lingual surface until plaster can be poured upon the surface which fits against the gum.

The thickness of rubber should then be reduced by the use of sharp scrapers and burs, and one or more grooves may be made wherever the plate needs strengthening, for the introduction of pieces of roughened German silver wire.

When the wire shall have been bent and prepared for the grooves referred to, narrow strips of rubber are packed into the grooves with a hot instrument, and the appropriate wire for each groove is warmed and pressed into the new rubber in the groove, the entire surface then being covered, wires and all, with rubber, piece by piece, to whatever thickness necessary, with the hot instrument.

This use of German silver wire in repairing fractured plates is particularly valuable in that it gives the required strength without the necessity of making the plate thick with rubber. Particularly is its use advisable in full dentures if the band be broken above the incisor teeth.

There is almost no limit to the use which may be made of this property of rubber, for it can be stuck to a plaster model with a hot instrument almost as well as to a basis of old rubber.

A few weeks ago a lady consulted me concerning a full upper rubber denture, the front teeth of which were too short, and as the plate had been worn a number of years, the suction was imperfect. There were reasons why it did not seem advisable to make a new plate, and as the lady had an older rubber plate which had been discarded some years before, I suggested that she allow me to experiment with that before doing anything to the one she was then wearing.

The laboratory man, under direction, first made a small groove all around the palatine surface near the edge of the plate, and packed into the groove with a hot instrument fresh rubber to form a ridge, or "Folsom," another one being made also around the air-chamber.

This surface of the plate was then invested in plaster and the six front teeth removed and replaced, two at a time, so as to be able to judge of their proper length and contour, the sockets being prepared for new rubber, which was packed in and the teeth lengthened as desired. The plate was then invested and vulcanized.

Although this plate had not been worn for at least five years, the suction was perfectly satisfactory fifteen minutes after it was put in the mouth, and while the "Folsom" ridge made the mouth sore and had to be partially scraped away at certain points two or three different times, within ten days the plate was as comfortable and as serviceable as a new one would have been.

This result being so satisfactory, the other plate was taken in hand and treated in the same manner except that the "Folsom" ridge was not made as high, and the lady did not return after the plate was put in the mouth, she sending me a note instead assuring me that the plate was comfortable and a success in every way.

Dr. J. N. Davenport, of Northampton, Mass., has made many partial rubber plates where he needs to use clasps, half-clasps, and small gold points to fit partially in between the teeth, without the use of a wax form. He takes a plaster impression, selects the teeth and grinds them to the model, fitting also the clasps and gold stays. The rubber is then packed directly upon the model with a hot instrument, the gold points, clasps, and teeth being placed accurately in position with the fingers, and rubber is packed about them as desired. The case is then invested and vulcanized, no opening of the flask or squeezing down being necessary, and much greater accuracy resulting.

No waxing up is necessary in any of these operations of addition or repair, the advantage being not only a great saving of time, but perfect union of new and old rubber and the ability to do in the hand accurately whatever is needed, eliminating all chance of malposition resulting from squeezing down in the flask.

I wish to impress upon my hearers that rubber packed in this way needs no undercut or dovetailed surface, a perfect union between the new and old rubber being obtained whenever the prepared surface of the old rubber is clean and dry.

Care should be taken not to touch the surface prepared for the new rubber, even with the fingers, for any interference with a freshly cut surface prevents perfect union.

Both the plate and the little pieces of new rubber should be at the temperature of the atmosphere, it being necessary to heat only the packing instrument.

Both the ordinary red and pink rubbers may be used as described above, but black rubber does not vulcanize well after the repeated application of a hot instrument.

